# Development and evaluation of a novel single nucleotide polymorphism panel for North American bison

**DOI:** 10.1111/eva.13658

**Published:** 2024-02-22

**Authors:** Sam Stroupe, James N. Derr

**Affiliations:** ^1^ Department of Veterinary Pathobiology, College of Veterinary Medicine Texas A&M University System College Station Texas USA

**Keywords:** conservation genetics, North American bison, population genetics – empirical, wildlife management

## Abstract

Genome‐wide single nucleotide polymorphism (SNP) genotyping platforms have become increasingly popular in characterizing livestock and wildlife populations, replacing traditional methods such as microsatellite fragment analysis. Herein, we report the development and evaluation of a novel bison SNP panel for population management and conservation. Initially, 2474 autosomal SNPs were selected from existing bison whole‐genome sequences and variable sites among bison on the GGSP bovine 50K Chip, based on minor allele frequency, data completeness, and chromosome location. Additionally, 20 mitochondrial SNPs were chosen to identify known mitochondrial haplotypes in bison according to previous research. The SNPs were further evaluated using genotyping‐by‐sequencing with 190 bison, representing the historical lineages that survived the major population crash of the late 1800s. Variants with high potential for genotyping error were filtered out, and the remaining SNPs were placed on a custom Illumina™ array. The final panel consisting of 798 autosomal and 13 mitochondrial SNPs was used to establish baseline genetic parameters, compare populations, and assign mitochondrial haplotypes in 995 bison across ten populations. These SNPs were also found to be highly informative for individual animal identification and parentage assignment. This SNP panel provides a powerful new method to establish a baseline for estimating genetic health of bison populations and a new tool for bison managers to make informed management decisions based on genetic information specific to their populations.

## 1 INTRODUCTION

During the 19th century, North American bison (*Bison bison*) underwent a significant population bottleneck and near extinction event (Hornaday, 1889). Species salvation was predicated by the establishment of five small private herds from wild captured calves and increased protection of the last two remaining wild populations. At the nadir of this population bottleneck, only these seven “founder” populations existed (Coder, 1975) with fewer than 1000 total animals. By the late 1800s, great efforts were made by both private bison owners and federal agencies to establish new conservation populations (American Bison Society, 1908a). However, with the limited number of animals available and very few protected areas to develop new populations, these populations were typically started with few individuals in geographically isolated areas.

Even today, most bison are found in small populations managed in isolation making them vulnerable to loss of genetic diversity and reduction in fitness though founder effects, random genetic drift, and inbreeding (Lacy, 1987). Therefore, long‐term management can greatly affect the conservation status and genetic health of these populations. One important aspect of population management includes monitoring genetic diversity, which provides valuable information for species conservation. Retaining genetic diversity should be an important factor in conservation goals as it can increase species survival success and genetic health (DeWoody et al., 2021; Forsman & Wennersten, 2016). Additionally, reduction in genetic diversity increases the risks for fertility problems and susceptibility to infectious diseases (Gibson, 2022). Considering the history of bison includes loss of genetic diversity through a massive population bottleneck and reliance of human‐mediated management for gene flow, genetic monitoring of populations is of great importance.

For the past two decades, microsatellite markers (STRs) have been the standard for genetic studies in North American bison including evaluation of population dynamics (Derr et al., 2011; Forgacs, 2019; Halbert et al., 2004, 2005; Halbert & Derr, 2008) and parentage testing (Schnabel et al., 2000). Though microsatellite‐based studies have proved a reliable method for parentage testing, animal identification, assessment of genetic diversity and population evaluation, the lowered cost of next‐generation sequencing has increased the use of single nucleotide polymorphisms (SNPs) in its place. Problems with microsatellite genotyping include differences in the technologies used to determine allele sizes, variation in dyes used to visualize DNA fragments and variations in the allele calling software which can contribute to inconsistent allele calls across laboratories (Vignal et al., 2002). In parentage assignment analysis and animal identification, discrimination power when using limited sets of microsatellites has proven particularly difficult in herds with highly skewed sex ratios, limited genetic variability, and high levels of inbreeding (Halbert et al., 2004). Such herds require additional microsatellite markers to accurately determine parentage which can be time consuming and expensive to the point of becoming impractical for large‐scale studies and routine testing. Additionally, SNPs offer many technical advantages over microsatellites such as easier automation (Anderson & Garza, 2006), lower mutation rates (Amorim & Pereira, 2005), and are less affected by inbreeding (Fernández et al., 2013).

The large number of loci, consistency, and ease of data sharing has increased the use of SNP‐based platforms for evaluating populations (Forcina & Leonard, 2020). High‐density SNP arrays designed for mammalian livestock have been used to evaluate the genetic health of closely related wildlife (Domínguez‐Viveros et al., 2023; Huisman et al., 2016; Stoffel et al., 2021). There have also been species‐specific SNP panels designed for wildlife to assist with species conservation goals and management such as wolves (Seddon et al., 2005), Iberian lynx (Kleinman‐Ruiz et al., 2017), and sable antelope (Gooley et al., 2020). In a case where SNPs were directly compared to microsatellites for the evaluation of sage grouse populations, SNPs revealed to be more effective in identifying population division than previously utilized microsatellite data (Zimmerman et al., 2020).

The use of SNP panels for parentage assignment has also been studied across a wide range of species, with the conclusion that SNPs are at least as powerful as microsatellites (Flanagan & Jones, 2019). SNP platforms have been proven as an effective method for parentage testing in several different mammalian species including European bison (Oleński et al., 2018; Tokarska et al., 2009), American bison (Yang et al., 2020), domestic cattle (Fernández et al., 2013; Heaton et al., 2002), sheep (Heaton et al., 2014), pigs (Lopes et al., 2013; Rohrer et al., 2007), and red deer (Gudex et al., 2014).

Here we present the first large‐scale genetic evaluation of North American bison populations using SNPs. A novel SNP panel was developed as a bison management tool for animal identification, genetic evaluation, and determining parentage in bison populations. Our aim was to use this SNP platform to: (1) establish a baseline to estimate the current genetic health of important bison populations in North America and (2) explore the use of this novel platform to provide a tool for bison managers to make informed management decisions based on genetic information specific to their populations.

## 2 METHODS

### 2.1 Biological material

**FIGURE 1:**
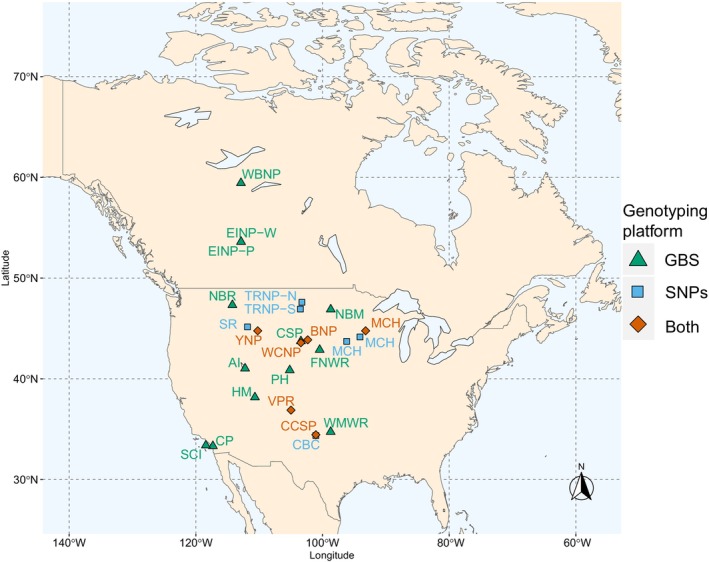
Map of sampled bison populations across North America. The genotyping platform, genotyping‐by‐sequencing (GBS), GGP Equine‐Bison SNP chip (SNPs), or both, used for each population is indicated by color and point shape. Number of samples used for each population and abbreviations can be found in Table 1.

North American bison samples included in this study were collected from 23 populations across 12 states in the United States of America, a Canadian province, and Canadian territory (Table 1, Figure 1). Hair samples from 192 North American bison (*Bison bison*) across 19 populations were used for variant filtering and validation using the genotyping‐by‐sequencing platform (Table 1). DNA was extracted from hair follicles according the Gentra Puregene kit (Qiagen) according to manufacturer protocol.

**TABLE 1:** Populations that were sampled for this study including population abbreviation, location, and sample size for each genotyping platform.

Population	Abbreviation	Location	GGP equine‐bison SNP chip samples	GBS samples	GGSP bovine 50K Chip samples
Antelope Island State Park	AI	Utah, USA	–	5	5
Badlands National Park	BNP	South Dakota, USA	57	10	–
Camp Pendleton Marine Corp Base	CP	California, USA	–	5	–
Caprock Bison Company	CBC	Texas, USA	68	–	–
Caprock Canyons State Park	CCSP	Texas, USA	177	28	4
Custer State Park	CSP	South Dakota, USA	–	8	2
Elk Island National Park (plains)	EINP‐P	Alberta, Canada	–	2	8
Elk Island National Park (wood)	EINP‐W	Alberta, Canada	–	2	8
Fort Niobrara National Wildlife Refuge	FNWR	Nebraska, USA	–	6	4
Henry Mountains	HM	Utah, USA	–	2	4
Minnesota Conservation Herd	MCH	Minnesota, USA	123	12	–
National Bison Range	NBR	Montana, USA	–	5	10
National Buffalo Museum	NBH	North Dakota, USA	–	2	–
Private Herd	PH	Colorado, USA	–	6	–
Santa Catalina Island	SCI	California, USA	–	4	–
Theodore Roosevelt National Park – North Unit	TRNP‐N	North Dakota, USA	48	–	–
Theodore Roosevelt National Park – South Unit	TRNP‐S	North Dakota, USA	50	–	–
Turner Enterprises Inc. – Snowcrest Ranch	SR	Montana, USA	68	–	–
Turner Enterprises Inc. – Vermejo Park Ranch	VPR	New Mexico, USA	66	33	5
Wichita Mountains National Wildlife Refuge	WMWR	Oklahoma, USA	–	6	4
Wind Cave National Park	WCNP	South Dakota, USA	66	42	–
Wood Bison National Park (wood)	WBNP	Northwest Territories, Canada	–	10	–
Yellowstone National Park	YNP	Wyoming, USA	272	2	9
	Total		995	190	63

Bison used in the evaluation of the SNP panel for genetic management include 995 hair or tissue biopsy samples were collected across ten populations (Table 1). Samples were sent to NeoGen for DNA extraction and genotyping. All bison sample used for this study were obtained during annual roundups or according to established management practices by the biologist/ranchers responsible for the management or ownership of the herds. All samples were collected by or under the direction of the owner, biologist, or ranch manager in charge of each bison herd. All samples genotyped with the GGP Equine‐Bison chip (NeoGen) represent modern populations. Collection years for each population are as follows: BNP 2019, CCSP 2021 & 2022, CBC 2021 & 2022, MCH 2021, SR 2020, TRNP 2016 & 2019, VPR 2021, WCNP 2018, YNP 2019 & 2021. Some samples for CCSP and MCH were collected earlier but are from bison confirmed to be in the population as of 2021.

### 2.2 Single nucleotide polymorphism (SNPs) panel selection

An initial set of 2474 autosomal SNPs were selected for this species‐specific SNP panel. Of these SNPs, 2077 were identified as biallelic SNPs from 25 previously generated bison whole‐genome sequences including samples from eight modern bison populations and five historical samples (Stroupe et al., 2022). SNPs from whole‐genome sequence data were selected according to the criteria: minor allele frequency (MAF) >0.3, <50% missing data, and a minimal distance of 1.2 Mbp apart with VCFtools v0.1.16 (Danecek et al., 2011). The remaining 397 SNPs were selected from the GGSP Bovine 50K Chip that had a minor allele frequency >0.1 and no missing data among a set of 63 genotyped bison (Table 1).

Selected SNPs were further evaluated using Allegro Targeted Genotyping platform (NuGEN Technologies, Inc). Of those, 1682 autosomal SNPs passed the probe design process for the genotyping‐by‐sequencing platform. In addition, 20 mitochondrial SNPs were included to distinguish intraspecific haplotype clades (Ward et al., 1999) and interspecific haplotypes of domestic cattle origin as described by Forgacs et al. (2016). 192 North American bison samples from 19 populations were genotyped at the 1702 SNPs that passed the probe design filtration using genotyping‐by‐sequencing (Table 1).

Genomic libraries were prepared according to the Allegro Targeted Genotyping user guide M01455 v3 (NuGEN Technologies, Inc) and sequenced using NovaSeq 6000 Illumina Next‐Generation Sequencing by Texas A&M Institute for Genome Sciences and Society (College Station, TX). Sequence data for each sample was then aligned to the Bos Taurus ARS‐UCD 1.2 reference genome using Burrows‐Wheeler Aligner, bwa‐mem v0.7.17 (Li & Durbin, 2009; Vasimuddin et al., 2019), and sorted using SAMtools v1.9 (Li et al., 2009). Variants were called using GATK v4.1.2.0 HaplotypeCaller, merged using the CombineGVCFs option, then genotyped using GenotypeGVCFs according to GATK best practices (DePristo et al., 2011; der Auwera & O'Connor, 2020; Poplin et al., 2017). Two samples were removed from the genotyping‐by‐sequencing dataset due to genotyping call rate below 50% before any filtering. Data filtration was done using VCFtools v0.1.16 (Danecek et al., 2011) or Plink v1.9 (Chang et al., 2015) according to the following criteria. The variant dataset was further filtered to include only biallelic SNPs with a minor allele frequency of at least 0.1, a Hardy–Weinberg equilibrium *p*‐values threshold of 0.005, sequencing depth >10, and <15% missing data resulting in 889 autosomal and 20 mitochondrial SNPs. The set of 909 SNPs was included on the GGP Equine‐Bison SNP Chip provided by NeoGen.

### 2.3 Autosomal single nucleotide polymorphism (SNPs) panel evaluation

Hair and biopsy tissue samples were sent directly to NeoGen where they were processed. DNA was extracted from samples according to NeoGen's standard procedures and genotyped according to standard Illumina Infinium HD Ultra Assay protocol guidelines on the GGP Equine‐Bison chip (NeoGen). Initial data quality control was done by NeoGen prior to the retrieval of the SNP data using Illumina's GenomeStudio with a call cutoff of 0.95.

The Illumina SNP Chip final report data was converted to plink lgen format using the script “illumina_to_lgen.R,” originally written by Ryan Schubert (github@RyanSchu), with adjustments specific to our dataset. In addition, a plink fam file was created from the sample list. The data were then converted into VCF format using Plink v1.9 (Purcell et al., 2007). Variants and samples with less than a 90% call rate were identified with VCFtools v0.1.16 (Danecek et al., 2011). During data quality control, 11 samples were identified and confirmed as duplicates, using the DuplicateCheck function in Sequoia v2.5.3 (Huisman, 2017). Each duplicate sample pair had less than a 1% genotyping error call rate. Low call rate and duplicate samples were then removed from further analysis resulting in a dataset of 798 SNPs and 995 samples. Allele frequencies revealed 64 monomorphic SNPs among the cohort of 995 samples. These SNPs were removed from the dataset for the following analyses, reducing the SNPs used to 734 autosomal SNPs.

Allele frequencies and inbreeding coefficients (*F*) were calculated using VCFtools v 0.1.16 (Danecek et al., 2011). Observed heterozygosity (H_O_), expected heterozygosity within populations (H_S_), allelic richness (A_R_), *F*_ST_, and *F*_IS_ with 95% confidence intervals were calculated with the Hierfstat R package (Goudet, 2005).Values calculated per individual or per loci were averaged across each population. These analyses were carried out in R v4.1.2 (R Core Team, 2022). Eigenvalues and eigenvectors were calculated by Plink v1.9 (Chang et al., 2015) and plotted in a principal component analysis (PCA) in R v4.1.2 (R Core Team, 2022) to evaluate similarity among bison individuals and populations. FastStructure v1.0 was used to determine population structure among all the bison sampled with values of *K* from 1 to 20 (Raj et al., 2014). The R package pophelper v2.3.1 (Francis, 2017) was then used to visualize and generate plots for the fastStructure results. The ‘chooseK.py’ function in fastStructure was used to test for the most likely number of populations represented from these data.

### 2.4 Parentage assignment

The VCF file was first converted to GenePop file format using PGDSpider v2.1.1.5 (Lischer & Excoffier, 2012). Cervus v3.0.7 (Kalinowski et al., 2007) was then used convert the GenePop file into a Cervus compatible input file. Calculation of non‐exclusion probabilities across all samples and 734 SNPs was done in Cervus v3.0.7 (Kalinowski et al., 2007). Sequoia v2.5.3 was used for parentage assignments due to the multi‐generational parentage assignment capabilities (Huisman, 2017). Pedigree construction was completed using two different datasets for Caprock Bison Company bison. The first was done using all 734 SNPs. In the second iteration, variants were filtered within this herd to only include SNPs with a minor allele frequency (MAF) >0.3, using Plink v1.9 (Chang et al., 2015) to retain only highly informative variants per software suggestion. The error rate was set to 0.01 on both runs but otherwise, default parameters were used. The reconstructed pedigree was then compared to the known pedigree based on cow‐calf pairings.

### 2.5 Mitochondrial assignment

In addition to the SNP filtration above, any mitochondrial SNPs that were heterozygous across 90% of samples were removed. Due to the haploid nature of mitochondrial genomes, it was assumed that these heterozygous sites were due to nuclear mitochondrial DNA (NUMT) segments or genotyping error. Mitochondrial haplotype assignments were made using 13 mitochondrial SNPs as described by Forgacs et al. (2016) that met the above criteria.

Each bison's mitochondrial haplotype was identified as either of bison or domestic cattle origin. Haplotypes of bison origin were further separated into major Clade I or II according to Forgacs et al. (2016). First, species‐specific haplotype assignment was made using ten SNPs that define bison or cattle mitochondrial DNA (mtDNA). Criteria were as follows, the sample species allele must be present for at least five of the ten SNPs with no alternative species alleles present. For example, if five or more bison alleles are present with no domestic cattle alleles, then the sample was assigned as having the bison mtDNA. Any sample that did not meet these criteria were labeled as ‘Unassigned’ for mtDNA species assignment.

The remaining three mtDNA SNPs we used to define interspecific clades. The criteria for clade assignments were similar to the species assignment in which, at least one SNP for the ‘Clade I’ allele must be present with no ‘Clade II’ alleles or vice versa. Samples that did not meet the criteria were labeled as ‘Unassigned’ along with any sample assigned the cattle or ‘Unassigned’ species haplotype. Any mtDNA SNP that was heterozygous for a sample was considered missing data and ignored in the assignments.

## 3 RESULTS

An initial set of 2494 SNPs were assessed for their use in genetic evaluation and management of bison populations. SNPs were selected from three sources, aligned whole‐genome sequence data, mitochondrial sequence data, and the GGSP bovine 50 K SNP chip. Probes were then designed for selected SNPs of which 1702 SNPs passed quality control for the design process. For further evaluation, a cohort of 190 bison that represent the major bison lineages and include most of the genetic diversity known to exist for this species was the genotyped using a genotyping‐by‐sequencing platform. After removing less informative SNPs and those with high potential for genotyping errors, a set of 909 SNPs was included on the GGP Equine‐Bison Illumina Infinium SNP Chip provided by NeoGen.

995 samples across ten populations were genotyped on this novel SNP panel using the designed SNP Chip. SNPs with a call rate below 90% were further removed from the final SNP panel resulting in 798 autosomal and 13 mitochondrial highly informative SNPs for evaluating indices of genetic diversity in North American bison. Of these SNPs, 64 were monomorphic among the cohort of bison genotyped thus far. While these SNPs were removed from the following analysis, based on our initial selection criteria, an alternative allele is present in the global bison population. Therefore, the potential that these SNPs are still informative exists. However, with the existing data, based on these 995 bison samples, we cannot recommend the use of these monomorphic SNPs for the evaluation of genetic diversity indices.

### 3.1 Population structure and differentiation

**FIGURE 2:**
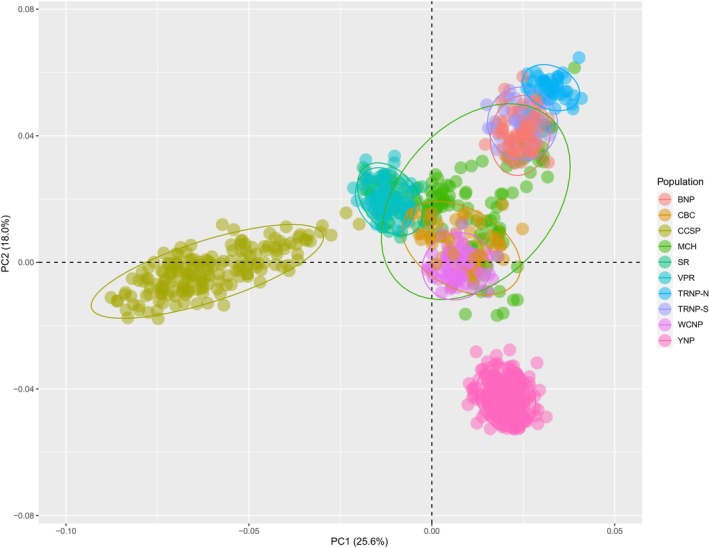
Principal component analysis (PCA) plot using 734 SNP from the GGP Equine‐Bison chip. Each population is identified by color with ellipses of 95% confidence level.

**FIGURE 3:**
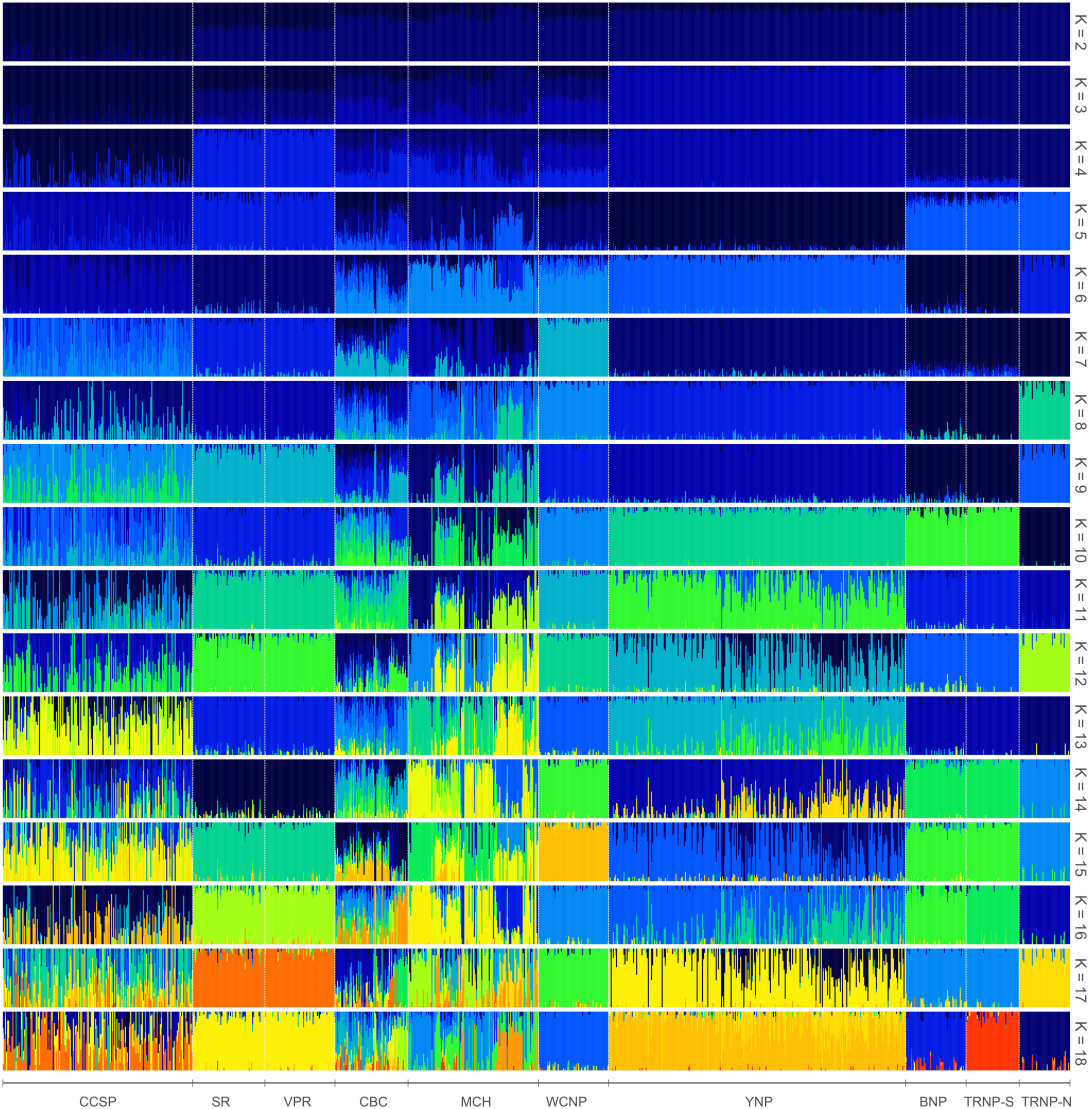
The fastStructure clustering plot for *K* = 2–18. Each vertical line represents individual bison grouped by population. Each horizontal bar represents a run of fastStruture at a different *K*. The model complexity that maximizes marginal likelihood equals 18 and the model components used to explain structure in the data is one. Plots were generated using pophelper v2.3.1 (Francis, 2017).

Insights of population structure and differentiation were exposed using principal component analysis (PCA) (Figure S1), fastStructure admixture analysis, and calculations of *F*_ST_ (Figures 2 and 3, Table 2). In the PCA, populations fall into five distinct clusters with Caprock Bison Co. (CBC) and the Minnesota Conservation Herd (MCH) overlapping with multiple clusters. The first cluster contains the Texas State herd at Caprock Canyons State Park (CCSP) which shows the most divergence from the other populations. The next cluster consists of both Turner Enterprise, Inc. (TEI) herds, Vermejo Park Ranch (VPR) and Snowcrest Ranch (SR), which are indistinguishable across all measures of population differentiation (*F*_ST_ = 0.003). The US National Park herds form the last three clusters with Yellowstone National Park (YNP) forming its own unit, Wind Cave National Park (WCNP) as a cluster with considerable overlap with CBC and the MCH, and the last cluster including Badlands National Park (BNP), Theodore Roosevelt National Park – South and North Units (TRNP‐S & TRNP‐N). However, TRNP‐N does show separation from BNP and TRNP‐S in both the PCA and fastStructure analysis between *K* = 6 and *K* = 8. BNP and TRNP‐S do not separate in the PCA but are distinguishable when *K* > 17. These identified clusters are also observed in the fastStructure analysis in *K* = 5. The model complexity that maximizes marginal likelihood, however, is at *K* = 18 with all populations though most clusters can be attributed to high levels of admixture observed in CCSP, CBC, and MCH. Each of the federal herds (BNP, TRNP‐N, TRNP‐S, WC, YNP) and the combined TEI herds are cohesive within populations in *K* = 1–20. Though at higher instances of *K* (*K* > 10), there is evidence of admixture in YNP. In almost all iterations of fastStructure CCSP, CBC and MCH all show substantial evidence of admixture, though not necessarily with other sampled populations. The highest differentiation among populations is between CCSP and TRNP‐N (*F*_ST_ = 0.249) and the lowest between the SR and VPR (*F*_ST_ = 0.003). Among the federal herds, WCNP and TRNP‐N have the highest differentiation (*F*_ST_ = 0.177) while BNP and TRNP‐S have the lowest (*F*_ST_ = 0.040). Largely, the analyses of population structure agree (Figure S2).

**TABLE 2:** Estimates of *F*_ST_ values for each population comparison as a measure of population differentiation.

Population	CCSP	SR	VPR	MCH	CBC	WCNP	YNP	BNP	TRNP‐S	TRNP‐N
CCSP		0.1427	0.1460	0.1535	0.1301	0.1561	0.1541	0.1938	0.2045	0.2492
SR	0.1427		0.0030	0.0990	0.0671	0.1172	0.1188	0.1398	0.1483	0.2038
VPR	0.1460	0.0030		0.1041	0.0730	0.1212	0.1248	0.1492	0.1581	0.2119
MCH	0.1535	0.0990	0.1041		0.0476	0.0704	0.0863	0.0814	0.0895	0.1248
CBC	0.1301	0.0671	0.0730	0.0476		0.0524	0.0591	0.0793	0.0876	0.1305
WCNP	0.1561	0.1172	0.1212	0.0704	0.0524		0.0860	0.1240	0.1344	0.1765
YNP	0.1541	0.1188	0.1248	0.0863	0.0591	0.0860		0.1152	0.1266	0.1642
BNP	0.1938	0.1398	0.1492	0.0814	0.0793	0.1240	0.1152		0.0403	0.0792
TRNP‐S	0.2045	0.1483	0.1581	0.0895	0.0876	0.1344	0.1266	0.0403		0.0884
TRNP‐N	0.2492	0.2038	0.2119	0.1248	0.1305	0.1765	0.1642	0.0792	0.0884	

### 3.2 Genetic diversity statistics

To characterize the genetic diversity within each population, *F*_IS_, average expected heterozygosity (H_E_), average observed heterozygosity (H_O_), average inbreeding coefficient (*F*), number of monomorphic SNPs, and average allelic richness were calculated using 734 biallelic autosomal SNPs for each population (Table 3). Mean *F*_IS_ values range from −0.0494 (MCH; 95% CI [−0.0674, 0.0426]) to 0.0018 (YNP; 95% CI [−0.0118, 0.0090]). TRNP‐N has the highest average inbreeding coefficient values (average *F* = 0.2656) and lowest average observed heterozygosity and allelic richness (H_O_ = 0.3196, A_R_ = 1.8855) whereas CBC has the lowest average inbreeding coefficient (*F* = 0.0069) and highest average observed heterozygosity (H_O_ = 0.4323), but YNP had the highest allelic richness (A_R_ = 1.9931).

**TABLE 3:** Estimates for measures of genetic statistics averaged across each population, based on 734 SNPs.

Population	Sample size	Mean Ho	Mean Hs	Mean *F*	Monomorphic SNPs	Mean AR	Mean *F*_IS_	Mean *F*_IS_ 95% confidence interval
CCSP	177	0.3689	0.3603	0.1501	29	1.9523	−0.0204	[−0.0374, −0.0117]
SR	68	0.3757	0.3701	0.1359	8	1.9790	−0.0091	[−0.0336, −0.0011]
VPR	66	0.3707	0.3655	0.1475	19	1.9648	−0.0109	[−0.0317, 0.0003]
MCH	123	0.4231	0.4012	0.0265	5	1.9892	−0.0494	[−0.0674, −0.0426]
CBC	68	0.4323	0.4259	0.0069	4	1.9927	−0.0114	[−0.0281, −0.0047]
WCNP	66	0.4018	0.3978	0.0747	5	1.9896	−0.0071	[−0.0239, 0.0017]
YNP	272	0.4202	0.4199	0.0341	3	1.9931	0.0018	[−0.0135, 0.0102]
BNP	57	0.3710	0.3664	0.1682	20	1.9664	−0.0065	[−0.0277, 0.0032]
TRNP‐S	50	0.3618	0.3550	0.1468	33	1.9462	−0.0155	[−0.0334, −0.0058]
TRNP‐N	48	0.3196	0.3175	0.2656	76	1.8855	−0.0029	[−0.0239, 0.0094]

### 3.3 Individual identity, parentage, & mitochondrial haplotype assignment

To evaluate usefulness in animal identification and parentage assignment, combined non‐exclusion probabilities were calculated for 734 autosomal SNPs with all 995 bison samples. The estimated individual identity (8.239E‐279), first parent (1.338E‐33), second parent (9.685E‐60), and parent pair (4.995E‐96) non‐exclusion probabilities suggest that this panel is highly informative and valid for individual identification and parentage verification. Moreover, estimated parentage assignments using this SNP panel were 100% concordant with 40 known cow‐calf pairings in the CBC population using the full set of 734 SNPs and a subset of 414 SNPs filtered for within population missingness, linkage, and minor allele frequency.

Additionally, 13 mitochondrial SNPs were able to individually assign haplotypes into intraspecific clades and interspecific mtDNA of bison or domestic cattle origin (Forgacs et al., 2016). Ten of which were able to identify species‐specific haplotypes to distinguish bison with domestic cattle or bison mitochondrial DNA and the remaining three distinguish the major mitochondrial clades found in bison. Of the samples genotyped on the SNP Chip platform, all had bison mtDNA. Most bison sampled were in Clade II including all MCH, WCNP, TRNP‐S, TRNP‐N, and the majority of CBC, BNP, and YNP samples. All samples from CCSP, VPR, and SR were included in Clade I along with a minority of CBC, BNP, and YNP samples (Table 4).

**TABLE 4:** Mitochondrial haplotype clade assignments percentages for each population. Assignments were made based off three clade defining SNPs (Forgacs et al., 2016).

Population	Sample size	Clade 1 (%)	Clade 2 (%)	Unassigned (%)
CCSP	177	100	0	0
SR	68	90	4	6
VPR	66	98	0	2
MCH	123	0	100	0
CBC	68	15	78	7
WCNP	66	0	95	5
YNP	272	35	65	0
BNP	57	16	84	0
TRNP‐S	50	0	100	0
TRNP‐N	48	0	100	0
Total	995	42	57	1

## 4 DISCUSSION

Since the near extinction event of bison in the late 19th century, survival and recovery of this species has relied almost entirely on human management. Through the efforts of many interest groups, bison have made a remarkable recovery to over 500,000 bison. Now bison are found across hundreds of populations with differing perspectives on bison management based on their primary goals whether it be conservation, livestock production, restoration, or education. Though these styles differ, many rely on genetic technologies to develop effective long‐term management strategies through routine genetic testing. Current methods rely on a core set of 12 nuclear microsatellite markers and two mitochondrial microsatellite markers (Schnabel et al., 2000; Ward et al., 1999). However, compared to SNPs, microsatellite markers are laborious, less informative, and inefficient for large‐scale studies which severely limits the amount of information that can be provided. Therefore, this presented novel SNP panel was developed as a resource for efficient evaluation of biodiversity across North American bison populations for the first time using genomic technologies and to provide a highly informative and cost‐effective tool for genetic management.

### 4.1 Population structure and differentiation

Each bison population has its own unique history that contributes to its modern genetic makeup. At the time when major bison populations were established, founder individuals were limited so populations typically started with small numbers in geographically isolated landscapes. As those populations grew, excess animals were subsequently used to start new herds or add to existing herds. Due to this, genetic exchange is reliant on anthropogenic dispersal that can be traced through historical records rather than assumptions from natural migration patterns like other wildlife species. This history of bison movements by humans between existing populations or in the establishment of new bison populations over the last 150 years certainly confounds genetic conservation studies and may result in misinterpreted or seemingly contradictory observations.

For example, the MCH is a collaborative effort to manage a statewide population comprised of several units, of which we have representatives from Minnesota Zoological Garden, Minneopa State Park, and Blue Mounds State Park included in this study. This population was initially started with bison from Fort Niobrara Wildlife Refuge (Nebraska, USA) but has since been supplemented with bison from other lineages (Minnesota Department of Natural Resources Division of Parks and Trails, 2016). An example of a recent bison herd establishment is the CBC herd. This herd was established in 2020 with bison cows from a private population of uncertain lineage and bulls from VPR. This recent admixture is reflected in both the PCA and fastStructure. Alternatively, the CCSP was established from decedents of Charles Goodnight's bison herd with recent introductions of bulls from VPR which can in part explain the observed admixture. VPR herd was started with YNP bison as well as other undocumented source populations.

All US National Park populations have well documented and interconnected histories (American Bison Society, 1908b; Coder, 1975; Stroupe et al., 2022). Briefly, YNP's population is derived from native animals, bulls from Charles Goodnight (TX, USA), and cows from Pablo and Allard (MT, USA) brought to the park in 1902 (Coder, 1975). WCNP was established with bison from Yellowstone and the New York Zoological Society. TRNP‐S was established with animals from Fort Niobrara Wildlife Refuge, which was in part established with Yellowstone animals. Bison from TRNP‐S were used to establish both the TRNP‐N and BNP. Although additional animals were later introduced into Badlands.

As expected, populations with recent introductions (CBC, CCSP, MCH) show higher levels of admixture in the fastStructure analysis and a larger distribution in the PCA. On the other hand, the Federal (BNP, TRNP‐S, TRNP‐N, WCNP, YNP) and Turner Enterprise Inc. (SR, VPR) populations have no recent introductions from unrelated populations which agrees with the tightly formed clusters and congruent admixture observed. The population structure and genetic distances show a clear separation between herds with clustering that was predicted by historical records and known relations (Figure S2).

### 4.2 Genetic diversity statistics

Evaluation of intrapopulation genetic diversity revealed that TRNP‐N has the lowest genetic diversity of the populations included in this study. This was also observed in a previous study conducted with microsatellites (Halbert & Derr, 2008). A likely contributing factor is serial founder effects leading to this population, which would be expected to cause a loss of genetic diversity (Nei et al., 1975). Additionally, drift is likely a contributing factor to the loss of genetic diversity due to the small starting population and lack of immigration into TRNP‐N (Lacy, 1987). When compared to its source, TRNP‐S, there is 4% reduction in average observed and expected heterozygosity. On the other hand, BNP which was also founded with animals from TRNP‐S has higher levels of heterozygosity and allelic richness. Opposed to TRNP‐N, BNP does not show similar signs of founder effect. An explanation for this observed pattern could be that in the establishment of these herds, BNP had a larger initial population and had additional introductions.

YNP, MCH, and CBC were on the higher end of estimated genetic diversity. YNP had the highest estimates of genetic diversity among the federal populations followed by WCNP. Although the sample size for YNP was larger, it was proportionally smaller given the census size of the herd, so that is unlikely a contributing factor in the analysis. YNP supports a population of 3000–6000 animals making it by far the largest population included in this study. A larger population, multiple founding sources, and wider geographic range could all be factors in the observed levels of genetic diversity and are important in the retention of that diversity. In previous comparisons of federal herds, YNP did not have the highest genetic diversity and was estimated as having slightly lower diversity as WCNP (Halbert & Derr, 2008). Differences in the findings of these studies are mostly likely attributed to sample selection strategies, resolution of markers, changes over time, or a combination of these factors. High genetic variability seen in MCH and CBC seem to be influenced by recent introductions of divergent animals.

The characterization of baseline genetic variability and inbreeding for populations is essential for understanding overall genetic health. With an establishment of current measures, future evaluations can directly compare to this study to identify and predict trends. Conservation plans can then be created or adjusted for each population. In the case of negative trends in genetic health, animals that would contribute the most to the recovery of that population could be identified using the presented resource. This could be vital for the management of small, isolated populations with high levels of inbreeding estimates and low genetic diversity.

### 4.3 Individual identity, parentage, & mitochondrial haplotype assignment

In addition, this SNP panel provides an efficient method for animal identification and parentage assignments which extends the utility of this panel. Managers will have reliable means to resolve practical issues such as having a consistent form of identification in cases of ownership disputes, insight on sire success, and maintaining pedigrees.

In small populations, such as many private bison herds across North America, it is necessary to document pedigrees to prevent negative impacts of inbreeding. While it may be relatively easy to determine cow‐calf pairs in small bison herds visually, identifying sires is only understood through genetic testing. In some cases, the true sire may be unexpected and could reveal insights into breeding structure. For example, at Wind Cave National Park parentage testing across 10 years using microsatellite data revealed that while sire success peaked at 6–8 years, around 10.7% of sires were only 1 year old at the time of breeding (Derr et al., 2011). This understanding of herd structure, allowed for improvements in genetic management programs and recommendations across similar populations. With a more efficient method of parentage testing, discernments such as this can be made across different management styles and populations where it was not feasible previously. This presented SNP panel will allow managers to maintain genetic diversity, prevent inbreeding, and understand herd dynamics in their populations.

The revelation that all bison included in this study have bison mtDNA is not surprising since many populations have selected against domestic cattle mtDNA due to negative phenotypic effects and potential for mitonuclear incompatibility (Derr et al., 2012). While mitochondrial DNA testing in bison has been a standard management practice for over a decade, this presented SNP panel has a higher resolution for population genetic studies and provides insight regarding species status and lineage assignment for maternal lineage mtDNA haplotypes. Testing of more populations will expand upon the finding of Forgacs et al. (2016) to further describe and define the distribution of mitochondrial diversity across bison.

### 4.4 Management implications

Here, we present the development and application of a novel genomics‐based tool for genetic management of North American bison. Sustained genetic monitoring is vital to preserving managed populations by identifying, predicting, and forming plans to document and conserve genetic diversity. This SNP‐based platform offers a streamlined approach to substantially increase the genetic testing productivity and provide higher resolution for various indices of genetic diversity as compared to previous technologies. With information more readily available, managers can make informed decisions based on current scientific technology. The application of these technologies will provide insight into the population and conservation genetics of bison that could well serve as a model for management of other private and public bison herds as well as other herd species.

## FUNDING INFORMATION

This research was supported by US DOI, National Park Service grant (P19AC00364). SS support was provided by a Throlson American Bison Foundation Scholarship, The Houston Safari Club and the College of Veterinary Medicine and Biomedical Sciences, Texas A&M University.

## CONFLICT OF INTEREST STATEMENT

The authors declare no conflicts of interest.

## Supporting information


Figure S1

Figure S2

## Data Availability

Data developed in this study are available from the authors.
