# Supplementary figures and images for: Development and evaluation of a novel single nucleotide polymorphism panel for North American bison
Source: Evol Appl. 2024 Feb 22;17(2):e13658. doi: 10.1111/eva.13658 (PMC10883761; doi:10.1111/eva.13658)

Scree Plot

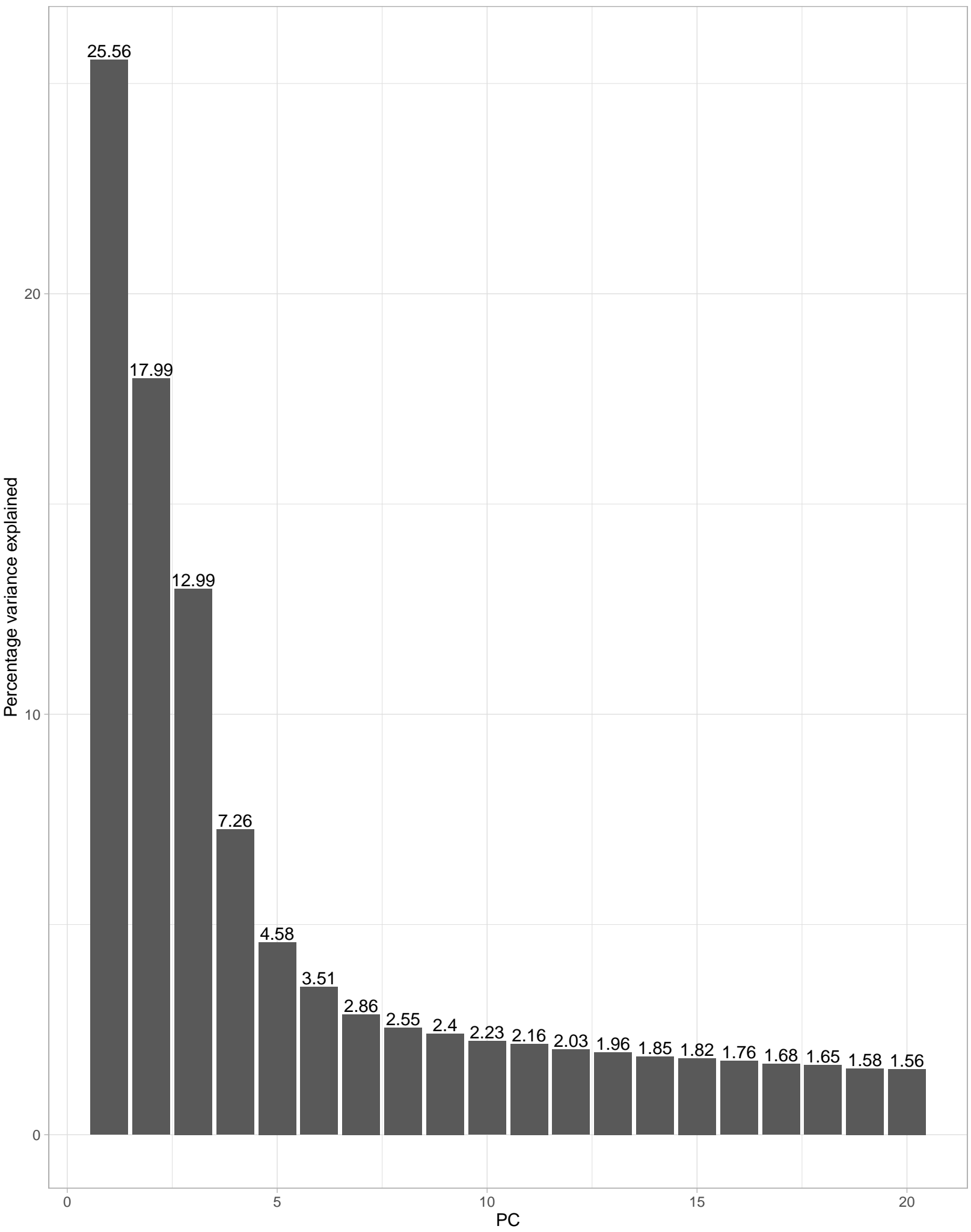

Supplement: Supplementary file 1 — Figure S1 [file EVA-17-e13658-s002.pdf]

Population

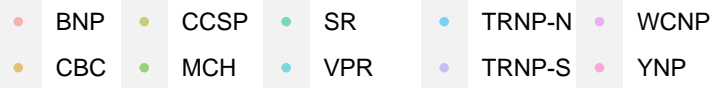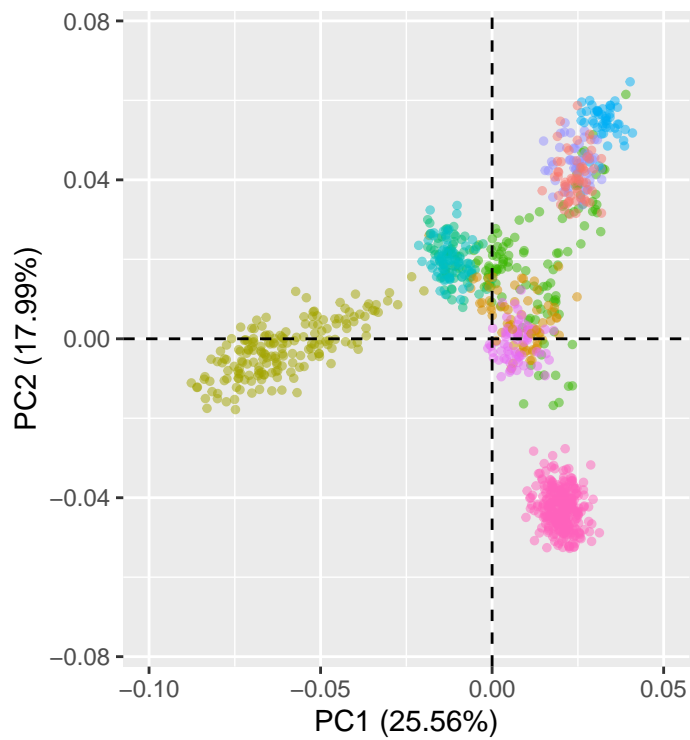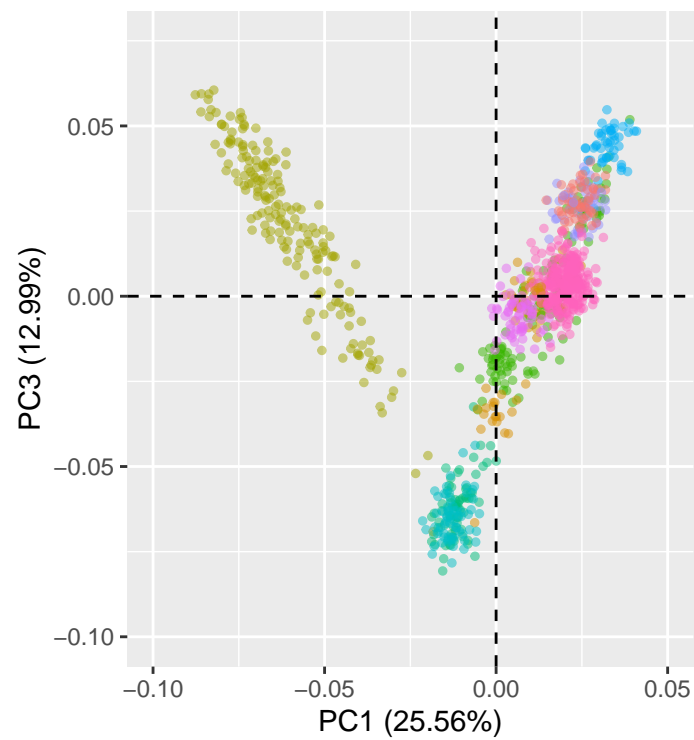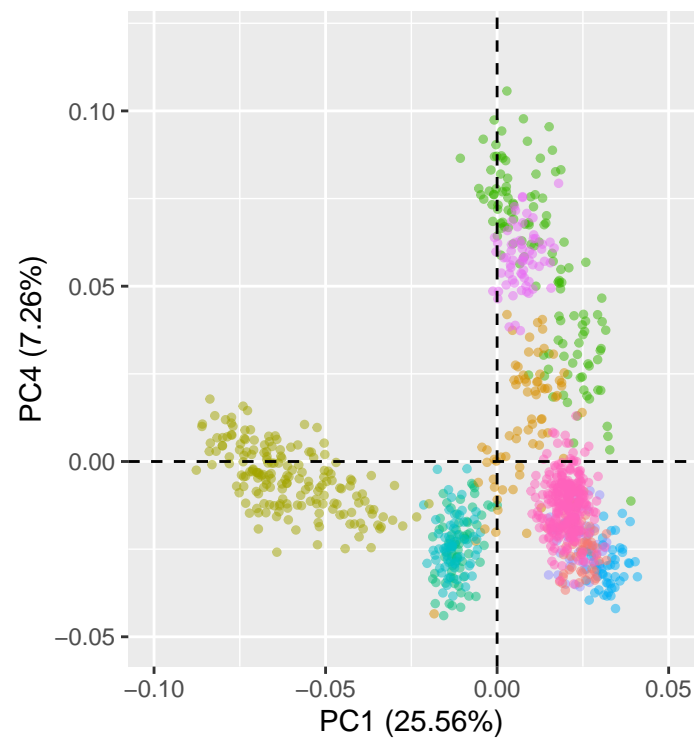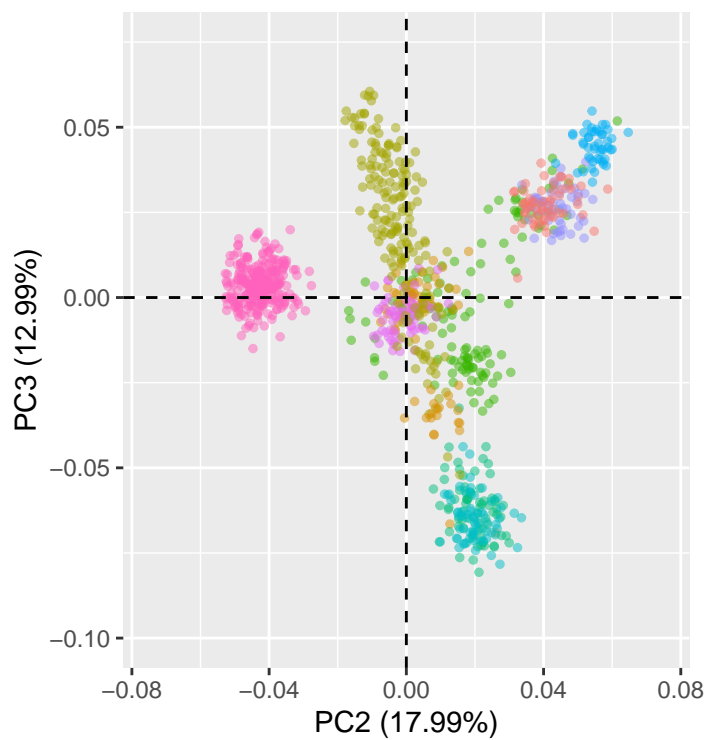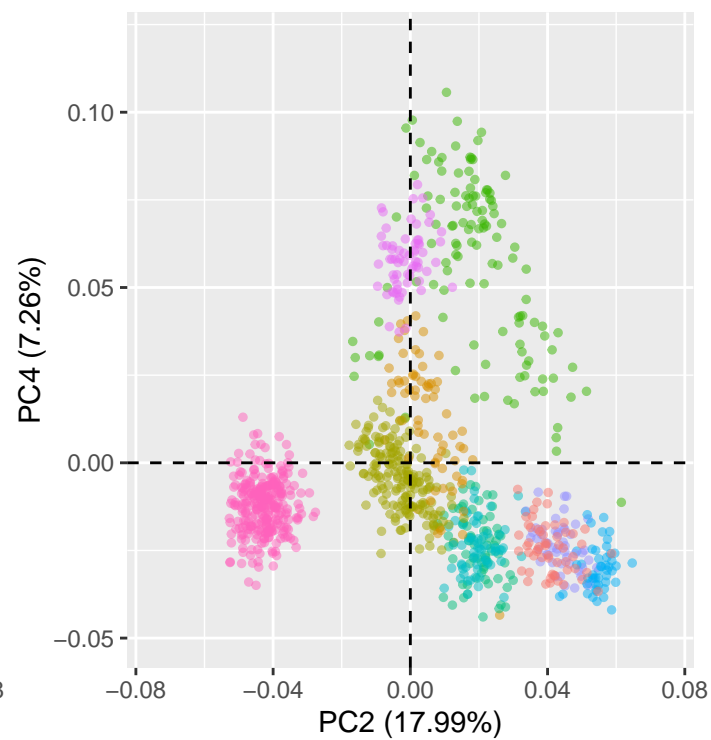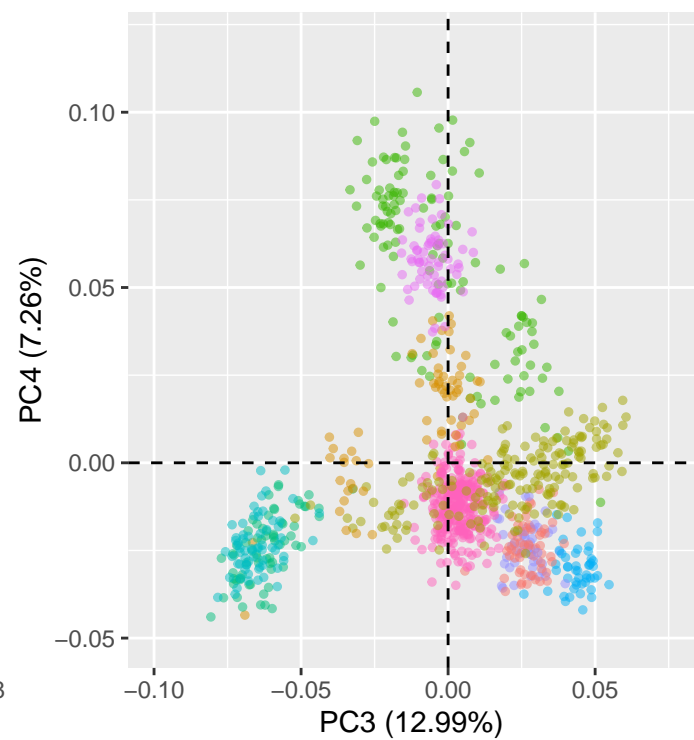

Supplement: Supplementary file 2 — Figure S2 [file EVA-17-e13658-s001.pdf]
